# Optimization of Preparation Process and Performance of Crumb Rubber/SBS-Composite-Modified Bitumen

**DOI:** 10.3390/ma19143041

**Published:** 2026-07-14

**Authors:** Renzhe Lei, Jianzhong Huang, Zhao Wang, Haojie Ji, Zunqing Liu, Jian Sun

**Affiliations:** 1 College of Transportation and Logistics Engineering, Xinjiang Agricultural University, Urumqi 830052, China; 2 Key Laboratory of Transportation and Logistics Engineering in Xinjiang, Urumqi 830052, China; 3 CCCC Second Highway Engineering Co., Ltd., Xi’an 710065, China

**Keywords:** crumb rubber/SBS composite modified bitumen, orthogonal design, response surface methodology, entropy-weighted TOPSIS method

## Abstract

To enhance the comprehensive performance of crumb rubber (CR)/SBS-composite-modified bitumen, an orthogonal experimental design was first adopted to analyze the effects of process parameters—including the addition sequence, shearing duration, shearing temperature and shearing speed—on the conventional properties of bitumen. Furthermore, based on the Box–Behnken response surface methodology, quadratic regression predictive models for the dosages of SBS, waste crumb rubber and aromatic oil were constructed, and the entropy-weighted TOPSIS method was introduced to comprehensively evaluate and multi-objectively optimize the proportioning schemes. The results indicate that a reasonable preparation process can significantly promote the uniform dispersion of modifiers, distinct non-linear interactions exist among the dosages of each modifier, and the optimal proportion balancing both high- and low-temperature performance was obtained through optimization. The constructed models exhibit good predictive accuracy, and the optimized CR/SBS-composite-modified bitumen showed excellent conventional properties and storage stability, which can provide a theoretical reference for the recycling of waste tires and the engineering application of composite-modified bitumen.

## 1. Introduction

With the continuous advancement of China’s strategy of building a strong transportation nation and the “the Belt and Road” initiative, high-grade highways are gradually developing towards heavy load and long service life [1]. At the same time, the combined effect of the continuous increase in traffic load and the extreme climate environment has put forward higher requirements for the service performance of asphalt pavement materials. Especially in typical continental arid climate regions such as Xinjiang, strong ultraviolet radiation, large temperature-difference cycles, low precipitation and heavy traffic have combined to make asphalt pavements face more severe service environments [2,3]. Taking the Hami region of Xinjiang as an example, the road surface temperature in summer can exceed 70 °C, and the minimum temperature in winter can drop to below −30 °C. The significant diurnal and seasonal temperature variations frequently induce distresses such as high-temperature rutting, low-temperature cracking and material aging [4]. In addition, due to the vast area of Xinjiang, modified bitumen often needs to undergo long-distance transportation and long-term high-temperature storage after production, and its performance stability directly affects pavement construction quality and service life [5]. Therefore, the development of high-performance modified bitumen materials with excellent road performance and engineering adaptability is of great significance for improving the service life of road infrastructure in Xinjiang and ensuring transportation safety.

Styrene-butadiene-styrene (SBS) block copolymers can form a continuous elastic network structure in bitumen, which can significantly improve the high-temperature rutting resistance, low-temperature crack resistance, and elastic recovery performance of the material [6,7,8,9], and have become one of the most widely used polymer modifiers. However, SBS-modified bitumen still has problems such as high modification cost, significant performance degradation after thermo-oxidative aging, and insufficient storage stability [10,11]. In contrast, bitumen modified with waste tire CR has advantages such as high resource recycling value, low cost, and excellent fatigue resistance, and shows broad application prospects in the field of green road materials [12,13]. However, CR-modified bitumen generally has defects such as high viscosity, high construction temperature, and easy segregation during storage [14,15,16].

To harness the complementary advantages of both modifiers, the CR/SBS composite modification technology has gradually become an important direction of modified bitumen research in recent years. By constructing a synergistic system of SBS elastic network-CR swelling enhancement, the high-temperature performance, low-temperature performance and economy of the material can be balanced, and the comprehensive road performance of composite-modified bitumen can be improved [17]. In this research field, scholars have carried out a lot of fruitful work. Studies have shown that introducing CR into SBS-modified bitumen can not only further enhance the high-temperature deformation resistance of the system through the oil absorption and swelling of CR particles; they suggest that the swollen CR particles also increase the viscosity of the continuous phase. This phenomenon is hypothesized to create a steric hindrance effect and a physically interwoven network with SBS, which effectively restricts the phase separation and coalescence of polymer particles, thereby significantly improving the storage stability of the composite-modified bitumen [18,19]. At the same time, the excellent elastic recovery ability of SBS can also effectively make up for the shortcomings of traditional bitumen in low-temperature ductility and easy cracking [20,21]. In addition, in response to the competition between CR and SBS for lightweight components in matrix bitumen and the problem of interfacial compatibility, existing studies have mostly adjusted the component balance by adding external admixtures such as aromatic oils and compatibilizers, or by using high-temperature and high shear, chemical activation, and other processes to promote the microscopic compatibility and synergistic dispersion of the two [22,23,24,25,26]. At present, research on CR/SBS-composite-modified bitumen mainly focuses on the optimization of modifier dosage, improvement in rheological properties, and microscopic mechanism of action. Existing studies have shown that the performance of the composite system is not only related to the dosage of SBS and CR, but also affected by a variety of factors such as the order of addition of the modifier, shear temperature, shear duration, shear speed, and type of additives [27,28]. However, at present, most studies still use single-factor experiments or controlled variable methods for analysis, which makes it difficult to reveal the interaction and non-linear coupling law between various factors [29]. At the same time, due to the complex swelling, adsorption, and compatibility between CR, SBS and light fractions, it is difficult to accurately reflect the comprehensive performance of the composite system by relying on a single performance index for ratio optimization [30]. In addition, most existing studies use the immediate performance after preparation as the evaluation basis [31], and pay insufficient attention to the structural evolution and performance change law of composite-modified bitumen during high-temperature storage, especially lacking systematic research on different thermal storage stages under engineering transportation and construction conditions [32,33]. In fact, in long-distance transportation areas such as Xinjiang, modified bitumen often needs to undergo a high-temperature storage process for several hours or even longer. During this period, the CR particles continue to absorb the light components in the bitumen and undergo further swelling, accompanied by a certain degree of degradation and component exchange. The SBS network structure will also undergo further development, reconstruction, and even local degradation, resulting in significant changes in the macroscopic properties of the system [34,35,36,37,38]. Therefore, the optimal ratio obtained based solely on short-term performance evaluation may not necessarily meet the actual engineering needs. Establishing a comprehensive optimization method that takes into account material composition, preparation process, and thermal storage evolution behavior is of great theoretical significance and application value for improving the engineering applicability of composite-modified bitumen.

To address the aforementioned gaps, a multi-stage sequential optimization framework was designed to prepare the CR/SBS-composite-modified bitumen. Initially, a comparative study and an orthogonal experimental design were employed to determine the optimal modifier addition sequence and key processing parameters [39]. Subsequently, the Box–Behnken response surface methodology (RSM) was utilized to model the interactive effects of modifier dosages on the conventional physical properties and storage stability of the composite system [40]. Crucially, to realistically simulate the dynamic performance evolution during short-term production and long-distance transportation in Xinjiang, two distinct thermal storage states (163 °C for 0.5 h and 24 h) were incorporated into the evaluation framework. Finally, an entropy-weighted TOPSIS method [41], coupled with experimental validation, was applied to identify the optimal formulation that synergistically balances multi-objective criteria, including thermomechanical resistance, storage stability, and workability.

Unlike previous studies that primarily focused on individual preparation parameters or single-stage material optimization, this research establishes a comprehensive optimization framework that simultaneously considers the preparation process, modifier composition, thermal storage evolution, and multi-objective performance evaluation. More importantly, the introduction of dual thermal storage states allows for quantitative assessment of the dynamic evolution of the CR/SBS composite system, while the entropy-weighted TOPSIS method provides an objective multi-criteria decision-making approach for selecting the optimal formulation under different storage conditions. Therefore, this study not only provides a reliable preparation strategy for CR/SBS-composite-modified bitumen suitable for the harsh climatic conditions of Xinjiang, but also offers a practical method for multi-factor synergistic optimization and engineering-oriented design of composite-modified bitumen.

## 2. Materials and Methods

### 2.1. Materials

The raw materials utilized in this study primarily included 60^#^ road petroleum bitumen (Xinjiang Chunfeng Tahe) as the base binder (Table 1) and a linear SBS modifier (Model T6302H, 30/70 block ratio, Xinjiang Dushanzi Petrochemical Co., Ltd., Karamay, Xinjiang, China) (Table 2). A 40-mesh crumb rubber (CR) derived from waste tires was selected as the secondary modifier, with its chemical attributes detailed in Table 3. Additionally, an aromatic oil refined via furfural extraction served as the processing additive. To facilitate cross-linking stabilization, elemental sulfur powder (purity ≥ 99.0%, Jinan Anchang Traffic Facility Co., Ltd., Jinan, Shandong, China) was introduced, the technical specifications of which are summarized in Table 4.

### 2.2. Sample Preparation

**Figure 1:**
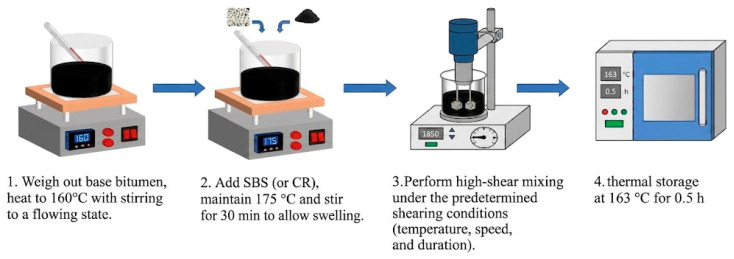
Schematic diagram of the sample preparation procedure.

The experiment was conducted by using a laboratory high-temperature and high-speed shear machine to prepare the samples. The preparation process is shown in Figure 1.

(1) The base bitumen was accurately weighed based on the blending ratio and heated to 160 °C until it reached a fully molten state.
(2) Modifiers and aromatic oil were added in a predetermined sequence. The system was then heated to approximately 175 °C and continuously stirred for 0.5 h to facilitate uniform swelling.
(3) The resulting blend was transferred to the high-speed shear mixer and subjected to high-shear compounding under prescribed temperature, speed, and duration. Afterward, the stabilizer was incorporated into the mixture, and shearing was sustained for another 1 h.
(4) Following the shearing stage, the composite-modified bitumen was conditioned in a 163 °C oven for 0.5 h of thermal development, guaranteeing thorough integration between the modifiers and the base bitumen. The first batch of specimen casting was then conducted. The bitumen was subsequently retained in the oven at 163 °C for an additional 24 h before the second batch of specimen fabrication was performed.

### 2.3. Comparative Experiment on Modifier Addition Sequence

In this study, a sequential optimization strategy was employed. To determine the optimal modifier addition sequence prior to the comprehensive optimization of processing parameters (Section 2.4) and material dosages (Section 2.5), a standardized baseline had to be established. To ensure maximum scientific rigor and logical consistency across the entire experimental framework, all controlled variables in this comparative phase were strictly fixed at the median levels of the subsequent experimental designs.

Specifically, the modifier dosages were established at the median levels of the Box–Behnken design (3 wt.% SBS, 15 wt.% CR, and 6 wt.% aromatic oil), while the preparation parameters followed the median levels of the orthogonal design (temperature of 190 °C, high-shear stirring at 4000 rpm, and duration of 60 min). Based on these unified baseline conditions, three distinct preparation protocols (Sequences A, B, and C) were developed to elucidate how the incorporation sequence affects the performance of the composite-modified bitumen.

The order of addition differs in the second step of Section 2.2. Sequence A: Heat the base bitumen to approximately 175 °C, add the SBS and aromatic oil, and then stir at a constant speed of 400 rpm for 0.5 h to allow it to fully swell; after that, add the CR and continue stirring for 0.5 h to achieve a uniformly dispersed and fully pre-swelled state.

Sequence B: After heating the bitumen, add CR and aromatic oil, stir at 400 rpm for 0.5 h to allow it to swell and desulfurize, and then incorporate SBS and continue stirring for 0.5 h to reach uniform dispersion.

Sequence C: After heating the bitumen, SBS, CR and aromatic oil are added together to the bitumen and stirred at 400 rpm for 1 h until evenly dispersed and fully pre-swollen.

Following the respective pre-swelling phases (Sequences A, B, and C), all mixtures proceeded to the high-shear mixing stage corresponding to the third step in Section 2.2. The entire blend was transferred to a high-shear mixer and subjected to shearing at a temperature of 190 °C and a speed of 4000 rpm for 60 min. Upon completion of this high-shear process, elemental sulfur was introduced as the cross-linking stabilizer, and the mixture was continuously sheared for an additional 1 h to finalize the preparation of the composite-modified bitumen.

According to the latest Standard Test Methods of Bitumen and Bituminous Mixtures for Highway Engineering (JTG 3410-2025 [42]), the standard testing temperature for the tube segregation test of high-viscosity modified bitumen, specifically rubber asphalt, is explicitly mandated at 180 °C. Therefore, to comply with the standard specifications, all segregation softening point difference evaluation tests in this study were conducted at 180 °C. It is crucial to clarify that this standardized 180 °C segregation test condition is fundamentally distinct from the 163 °C thermal storage process discussed in subsequent sections. The 163 °C thermal storage is an engineering simulation designed to replicate the bulk storage and long-distance transportation environment within heated tank trucks, which is particularly relevant for practical pavement construction in vast geographic regions like Xinjiang. By strictly separating the standard testing temperature (180 °C) from the engineering transport simulation temperature (163 °C), the performance variations of the composite-modified bitumen arising from these different structural development pathways were systematically characterized via critical indicators—namely penetration, softening point, ductility, and segregation softening point difference—thereby establishing the optimal modifier addition sequence.

### 2.4. Fabrication Process Optimization via Orthogonal Design

Following the determination of the optimal modifier addition sequence, the fabrication process was systematically optimized. Shearing duration, shearing temperature, and shearing speed were selected as the primary controlling factors. A three-factor, three-level orthogonal experimental design comprising nine distinct runs was implemented. The specific factor levels, established through preliminary experiments, are detailed in Table 5, while the complete experimental matrix is presented in Table 6. All experiments were conducted in triplicate, with the average values and standard deviations reported. The resulting CR/SBS-composite-modified bitumen samples were characterized based on their physical properties (penetration, softening point, and ductility) and storage stability (segregation softening point difference). Finally, range analysis was employed to evaluate the significance of each factor’s impact on the binder performance, thereby identifying the optimal combination of processing parameters.

### 2.5. Response Surface Methodology (RSM) Experimental Design

A Box–Behnken Design (BBD) was employed for experimental design and optimization analysis. SBS dosage (A), CR dosage (B), and aromatic oil dosage (C) were selected as the independent variables, with ranges of 2–4 wt.%, 10–20 wt.%, and 2–10 wt.%, respectively. The factor ranges were determined based on published literature, preliminary single-factor trials, and engineering feasibility. The selected levels covered the practical composition ranges reported in previous studies while ensuring adequate processability, avoiding excessive viscosity and modifier agglomeration when the combined SBS and CR dosage exceeded approximately 25 wt.%. (expressed as weight percentages relative to the base bitumen). Penetration, ductility, softening point, and softening point difference were chosen as the response variables to characterize the workability and flow behavior, low-temperature cracking resistance, high-temperature stability, and storage stability and homogeneity of the composite-modified bitumen, respectively. The factors and levels used in the response surface experiments are presented in Table 7. Each experiment was conducted in triplicate, and the average values together with the standard deviations were reported. Based on the experimental results, regression models were developed for each response variable. Analysis of variance (ANOVA) was utilized alongside RSM to systematically investigate the main and interactive effects of these factors on the composite-modified bitumen, ultimately determining the optimal modifier combination.

### 2.6. Entropy-Weighted TOPSIS Comprehensive Evaluation

The performance of the CR/SBS-composite-modified bitumen is governed by complex multi-factor interactions, often resulting in trade-offs among individual indicators. Because relying on a single metric cannot capture the holistic properties of the material, a multi-criteria decision-making approach is required. To verify the RSM optimization results and ascertain the definitive optimal formulation under both 0.5 h and 24 h thermal storage conditions, an entropy-weighted TOPSIS (Technique for Order Preference by Similarity to Ideal Solution) model was introduced.

Unlike conventional subjective weighting methods, the entropy-weighted approach calculates index weights objectively based on the information entropy of the experimental data, thereby eliminating artificial bias. The TOPSIS algorithm then ranks the candidate formulations by measuring their geometric distances from both the positive-ideal and negative-ideal solutions. This integrated method allows for a robust, multi-objective evaluation of the binder across varying degrees of engineering importance (i.e., penetration, ductility, softening point, and softening point difference). The specific calculation steps are as follows [43].

(1) Construction of the initial evaluation matrix:

(1) $$X\;=\;{\left(x_{ij}\right)}_{m\times n}$$

where m denotes the number of evaluation alternatives, n represents the number of evaluation indicators, $x_{\mathrm{ij}}$ signifies the empirical value of the j-th indicator corresponding to the i-th alternative, and X is the initial evaluation matrix.

(2) To eliminate the scaling effects stemming from discrepancies in dimensions and variation ranges among various indicators, the min–max normalization method was employed to transform the raw data into a dimensionless format.

For benefit (positive) indicators,

(2) $$r_{\mathrm{ij}}=\frac{X_{\mathrm{ij}}-X_{\min}}{X_{\max}-X_{\min}}$$

For cost (negative) indicators,

(3) $$r_{\mathrm{ij}}=\frac{X_{\max}-X_{\mathrm{ij}}}{X_{\max}-X_{\min}}$$

For nominal-the-best (moderate) indicators, such as penetration,

(4) $$r_{\mathrm{ij}}=1-\frac{\left|X_{\mathrm{ij}}-X_{0}\right|}{\max\left|X_{\mathrm{ij}}-X_{0}\right|}$$

where $X_{0}$ represents the median value of the targeted optimal range.

Upon completing the indicator standardization, the weight of each indicator was determined via the entropy weight method, based on which the weighted normalized decision matrix was constructed:

(5) $$V\;={\left(\omega_{j}r_{\mathrm{ij}}\right)}_{m\times n}$$

where $\omega_{j}$ represents the weight assigned to the j-th indicator.

(3) Determination of the positive-ideal and negative-ideal solutions from the normalized decision matrix, followed by the calculation of the Euclidean distances between each alternative and these ideal solutions:

(6) $$\left\{\begin{array}{c} V^{+}\;=\;\left[\max\left(v_{i1}\right)\right]\;=\;\left[v_{1}^{+},\;v_{2}^{+},\;\cdots ,\;v_{n}^{+}\right]\\ V^{-}\;=\;\left[\min\left(v_{i1}\right)\right]\;=\;\left[v_{1}^{-},\;v_{2}^{-},\;\cdots ,\;v_{n}^{-}\right] \end{array}\right.$$

where $V^{+}$ and $V^{-}$ denote the positive-ideal and negative-ideal solutions, respectively.

(7) $$\left\{\begin{array}{c} D_{i}^{+}=\sqrt{\sum_{j=1}^{n}{\left(v_{\mathrm{ij}}-v_{j}^{+}\right)}^{2}}\\ D_{i}^{-}=\sqrt{\sum_{j=1}^{n}{\left(v_{\mathrm{ij}}-v_{j}^{-}\right)}^{2}} \end{array}\right.$$

where $D_{i}^{+}$ and $D_{i}^{-}$ represent the Euclidean distances of the i-th alternative from the positive-ideal and negative-ideal solutions, respectively.

(4) Calculation of the comprehensive closeness coefficient for each alternative:

(8) $$C_{i}\;=\;\frac{D_{i}^{-}}{{D_{i}^{+}\;+\;D}_{i}^{-}}$$

where a value of $C_{i}$ closer to 1 signifies that the corresponding alternative is closer to the optimal ideal state, thereby indicating superior comprehensive performance.

Compared with the conventional TOPSIS method, the entropy-weighted TOPSIS approach determines the weight of each evaluation index objectively according to the information entropy of the experimental data, thereby avoiding subjective bias caused by artificial weight assignment. Considering that penetration, ductility, softening point and storage stability exhibit different degrees of variation and engineering importance, the entropy-weighted TOPSIS method was adopted to comprehensively evaluate the overall performance of the CR/SBS-composite-modified bitumen and to identify the optimum formulation under multiple performance objectives.

## 3. Results and Discussion

### 3.1. Influence of Modifier Addition Sequence on Composite System Performance

**Figure 2:**
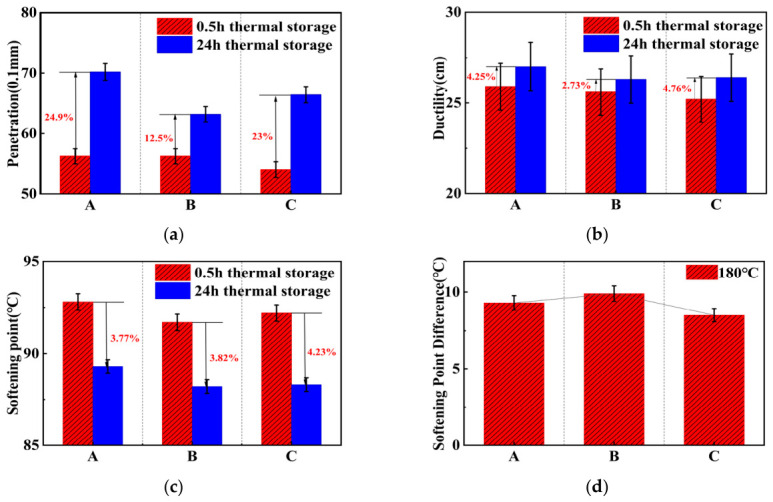
Schematic illustration of the impacts of three feeding sequences. (**a**) Influence of feeding sequence on penetration. (**b**) Influence of feeding sequence on ductility. (**c**) Influence of feeding sequence on softening point. (**d**) Influence of feeding sequence on softening point difference.

As delineated in Figure 2, the CR/SBS-composite-modified bitumen fabricated via different modifier feeding sequences all satisfy the specification criteria. Specifically, the penetration values fall within the range of 50–70 (0.1 mm), the ductility values exceed 20 cm, and the softening points are well above 70 °C. These indices demonstrate that all three incorporating methods achieve effective modification of the base bitumen through the combined action of CR and SBS. Nevertheless, distinct variations emerge regarding the performance retention capacities of the composite systems following high-temperature storage under different adding sequences.

As illustrated in Figure 2a–c, after thermal conditioning at 163 °C for 24 h, the conventional physical properties of the three groups evolved to varying degrees yet remained at a commendable level. The penetration of each group exhibited an upward trend, with Groups A and C showing pronounced increments—rising from 56.2 and 54.0 (0.1 mm) to 70.2 and 66.4 (0.1 mm), representing surges of 24.9% and 23.0%, respectively. Conversely, Group B experienced a minimal increase of only 12.5%. This phenomenon implies that elevated-temperature storage induces a certain extent of structural degradation and network relaxation within the modifiers. Correspondingly, a marginal increase was observed in the ductility of all groups (with increments of 4.76%, 4.25%, and 2.73% for Groups C, A, and B, respectively), which is attributed to the continuous maltene absorption and swelling of CR particles that inherently bolstered the low-temperature flexibility of the system. Concurrently, although the softening points marginally declined due to the localized weakening of the modified networks—decreasing by 3.77% (from 92.8 °C to 89.3 °C for Group A), 3.82% (Group B), and 4.23% (Group C)—the residual softening points consistently stayed above 88 °C, highlighting outstanding high-temperature stability. The variation in the segregation softening point difference is shown in Figure 2d. Under the 163 °C storage regime, the softening point differences between the top and bottom of the tube for Groups A, B, and C were 1.9 °C, 2.2 °C, and 1.3 °C, respectively. When the storage temperature escalated to 180 °C, these softening point differences significantly widened to 9.3 °C, 9.9 °C, and 8.5 °C. Although intensified phase separation occurred in the systems at elevated temperatures, Group C consistently maintained the lowest softening point difference, proving that the simultaneous incorporation process can effectively suppress modifier stratification and optimize storage stability. While the softening point difference index of Group A was slightly higher than that of Group C, its absolute softening point difference value strictly complied with the storage stability thresholds specified in the JTG 3410-2025 standard for modified bitumen.

From a mechanistic perspective, the Group A process (SBS-prioritized) is hypothesized to allow SBS to initially establish a continuous elastomeric network within the base bitumen. The subsequently added CR particles are then likely anchored within this polymeric skeleton to undergo further swelling, yielding a potentially synergistic system. In Group B (CR-prioritized), the initial addition of CR is thought to deplete a significant portion of light components, which may severely inhibit the subsequent swelling and network development of SBS. In Group C (simultaneous addition), the co-existence of SBS and CR likely triggers competitive swelling, thereby restricting the collaborative manifestation of the modification effects.

To rigorously justify the selection of Group A over Group C, a data-driven evaluation focusing on base thermomechanical robustness was applied. At the mandated 180 °C extreme test temperature, intense phase separation occurred across all preliminary groups, resulting in massive softening point differences (9.3 °C for Group A and 8.5 °C for Group C). Because all unoptimized preliminary sequences significantly exceeded the standard threshold at this extreme temperature, the marginal 0.8 °C advantage of Group C cannot serve as the decisive metric. Instead, absolute structural retention becomes the primary selection criterion. For pavements operating in severe continental arid climates, high-temperature network stability is paramount. Group A demonstrates a quantitatively superior polymer network, achieving absolute softening points of 92.8 °C and 89.3 °C after 0.5 h and 24 h of thermal conditioning, respectively. After 24 h of extreme heat, Group A’s softening point only degraded by 3.77%, compared to a more severe structural degradation of 4.23% in Group C. Furthermore, Group A consistently yielded higher absolute ductility values. Therefore, Sequence A (SBS-prioritized) was definitively selected to establish a superior performance baseline. The mitigation of the high-temperature segregation issue was strategically deferred to the subsequent orthogonal and Box–Behnken optimizations (Section 2.4 and Section 2.5), where shearing parameters and modifier dosages were systematically fine-tuned to eventually satisfy the storage stability thresholds.

### 3.2. Orthogonal Experimental Results and Analysis

#### 3.2.1. Experimental Results

**Figure 3:**
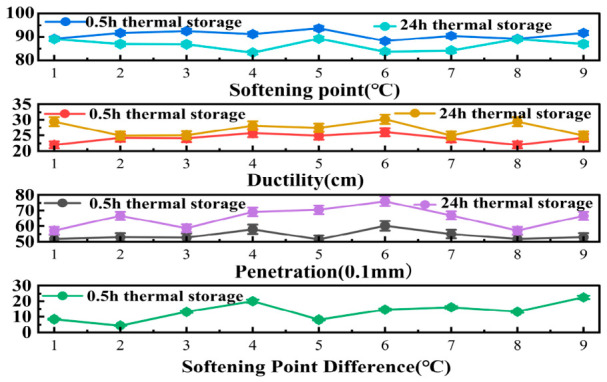
Orthogonal experimental data.

The CR/SBS-composite-modified bitumen specimens were prepared according to the prescribed orthogonal experimental design. To systematically characterize the performance evolution, the conventional physical properties and storage stability were evaluated following thermal conditioning at 163 °C for 0.5 h and 24 h. The resulting performance indices are summarized in Figure 3.

As illustrated in Figure 3, the processing parameters significantly influence the macroscopic performance of the composite binder. Variations in shearing parameters induced distinct fluctuations in the softening point, ductility, and 180 °C segregation softening point difference. Mechanistically, appropriate shearing conditions are hypothesized to facilitate the uniform dispersion and adequate swelling of SBS and CR within the base matrix, thereby fostering a stable composite architecture. In contrast, insufficient shearing energy likely results in non-uniform modifier dispersion, which compromises the overall performance of the system. Conversely, excessive shearing temperatures or prolonged durations may exacerbate the volatilization of light fractions and trigger the structural degradation of the polymer networks, ultimately undermining modification efficacy. Therefore, a quantitative range analysis is required to evaluate the statistical significance of each factor and identify the optimal processing parameters.

#### 3.2.2. Range Analysis

**Figure 4:**
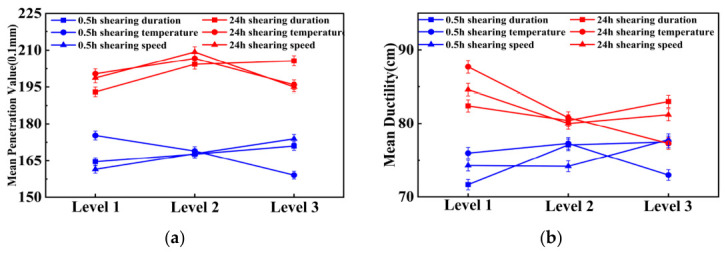

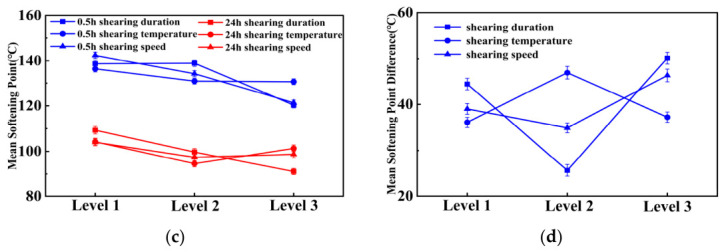
Mean values of various factors at different levels. (**a**) Range analysis results for penetration. (**b**) Range analysis results for ductility. (**c**) Range analysis results for softening point. (**d**) Range analysis results for softening point difference.

**Figure 5:**
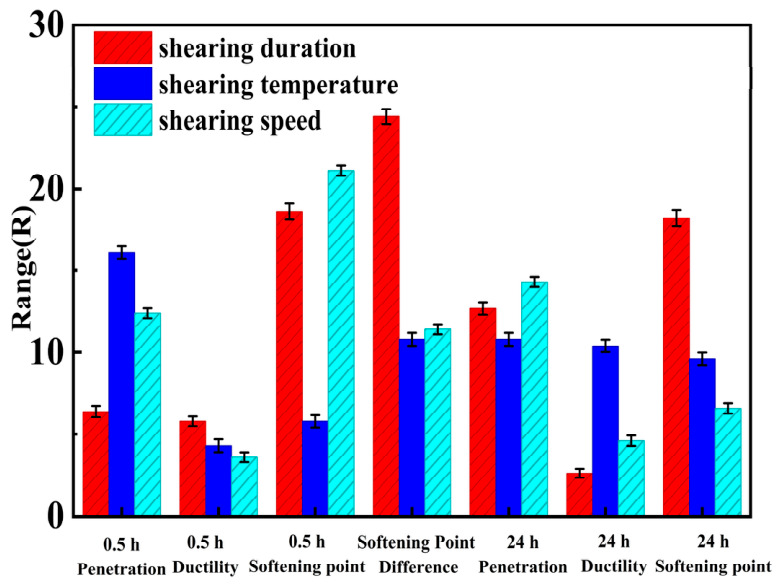
Experimental results of range values (R).

Figure 4 and Figure 5 present the mean experimental responses at varying factor levels alongside the range (R) analysis outcomes. The R value directly reflects the sensitivity of a performance metric to changes in a specific processing parameter. Variations in these dominant factors across different thermal storage periods provide indirect insights into the internal structural evolution of the composite bitumen. Since the range analysis reflects the sensitivity of each response to changes in the processing parameters, variations in the dominant factors under different thermal storage periods also provide insight into the evolution of the internal structure of the CR/SBS composite bitumen. Therefore, the R values are discussed not only from a statistical perspective but also in terms of the corresponding swelling, dispersion, compatibility, and thermal degradation mechanisms.

The range analysis indicates that the performance metrics of the composite-modified bitumen exhibit distinct time-dependent and multi-factor synergistic characteristics. During the initial storage stage, elevated shearing temperatures are hypothesized to accelerate the diffusion of light fractions into the CR particles, likely promoting rapid swelling and facilitating the extension of SBS molecular chains. This dynamic is thought to establish a relatively continuous polymer network. However, excessively high temperatures may simultaneously trigger thermal oxidation and chain scission of SBS, leading to partial network degradation.

Specifically, the shearing temperature exerts the most pronounced impact on penetration; elevated temperatures likely accelerate the diffusion of light fractions into the CR core while potentially inducing slight polymer degradation, thereby altering the system’s rheology. Meanwhile, higher shearing speeds are believed to enhance mechanical dispersion homogeneity, playing a predominant role in elevating the softening point. Furthermore, an appropriate shearing duration is hypothesized to provide sufficient interaction time for physical cross-linking between SBS and CR, thus benefiting low-temperature ductility. During prolonged (24 h) thermal storage, the migration of light components gradually approaches equilibrium. At this stage, CR particles are presumed to undergo secondary swelling by absorbing surrounding aromatic fractions. Concurrently, the thermodynamic relaxation of the SBS network and potential degradation of polymer chains may reduce structural constraints on the swollen CR particles. This explains why phase compatibility becomes increasingly dependent on the aromatic oil dosage after 24 h of storage [44,45]. Notably, the influence of shearing speed on penetration increases significantly during this extended period, highlighting its critical role in facilitating secondary modifier dispersion. Conversely, excessive shearing durations and temperatures may induce mechanical scission of SBS macromolecules and accelerate light-component volatilization. The resulting reduction in network integrity is likely responsible for weakened elastic recovery, ultimately manifesting as diminished ductility and softening point values.

In summary, the macroscopic performance evolution of the CR/SBS-composite-modified bitumen appears to be governed by the complex interplay of CR swelling, SBS network formation and relaxation, and light fraction redistribution. As these microstructural mechanisms continuously compete throughout the preparation and storage processes, the dominant processing parameter governing the material’s performance shifts accordingly over time.

#### 3.2.3. Optimization Conclusions of Processing Parameters

Synthesizing the evolution profiles of penetration, softening point, ductility, and softening point difference under the 0.5 h and 24 h thermal storage conditions, it is evident that the performance of the CR/SBS-composite-modified bitumen is highly sensitive to variations in shearing duration, shearing temperature, and shearing speed. The range analysis results presented in Figure 5 further demonstrate that the dominant factor varies with both the performance indicator and the thermal storage period. During the short-term thermal development (0.5 h), the shearing temperature exhibits the largest range value for penetration (R = 16.1), indicating that it primarily governs the initial swelling behavior of CR and the formation of the SBS network. The shearing speed has the greatest influence on the softening point (R = 21.1), whereas the shearing duration plays the dominant role in improving ductility (R = 5.8) and reducing softening point difference (R = 24.4). After 24 h of thermal storage, the controlling factors shift owing to the continuous swelling and structural evolution of the composite system. Specifically, the influence of shearing speed on penetration becomes more pronounced (R = 14.3), while shearing temperature dominates ductility (R = 10.4), and shearing duration remains the most significant factor affecting both the softening point (R = 18.2) and softening point difference (R = 24.4). These results indicate that an appropriate processing protocol can effectively suppress the deterioration of the bitumen properties during prolonged thermal storage.

Based on the combined evaluation of the mean response plots (Figure 4) and the range analysis (Figure 5), the optimal processing parameter combination was identified as A_2_B_1_C_2_, corresponding to a shearing duration of 60 min, a shearing temperature of 185 °C, and a shearing speed of 4000 rpm. This combination provides a balanced optimization of high-temperature performance, low-temperature ductility, and storage stability, while simultaneously ensuring that all conventional performance indices satisfy the specification requirements. Therefore, it was selected as the optimal processing parameter combination for the subsequent response surface optimization and mixture proportion design.

### 3.3. Optimization of Modifier Dosages Using Response Surface Methodology

#### 3.3.1. Data Fitting and Model Construction

With the SBS dosage (A), CR dosage (B), and aromatic oil dosage (C) designated as the independent variables, and penetration, ductility, softening point, and softening point difference selected as the responses, the response surface experimental results under thermal storage durations of 0.5 h and 24 h were obtained, as detailed in Table 8. Second-order (quadratic) polynomial regression fitting was separately performed on the two sets of experimental data to construct predictive models for the performance of the composite-modified bitumen. Concurrently, the overarching influences of the 0.5 h and 24 h high-temperature storage horizons on the bitumen properties were systematically analyzed.

To further evaluate the adequacy of the developed response surface models, the statistical indicators obtained from the ANOVA were comprehensively examined. The results demonstrate that all developed regression models are statistically significant (*p* < 0.05) and possess satisfactory fitting accuracy and predictive capability. Moreover, the relatively close agreement among the R^2^, Adjusted R^2^, and Predicted R^2^ values indicates that the established models exhibit good robustness and generalization ability within the investigated experimental domain.

To improve the interpretability and predictive reliability of the models, regression terms that were statistically insignificant (*p* > 0.05) were eliminated while preserving the hierarchical structure of the quadratic polynomial models. Consequently, the final reduced quadratic regression equations retained only the significant linear, interaction, and quadratic terms, enabling the models to accurately characterize the non-linear relationships between the preparation variables and the performance responses. Therefore, the developed response surface models provide a reliable mathematical framework for describing the synergistic effects of SBS dosage, CR dosage, and aromatic oil dosage on the conventional properties of CR/SBS-composite-modified bitumen and provide a sound theoretical basis for the subsequent response surface analysis and optimization.

#### 3.3.2. Model Analysis

Penetration is primarily employed to characterize the consistency and high-temperature flow behavior of composite-modified bitumen, with its magnitude directly reflecting the capability of materials to resist permanent deformation under elevated temperatures. Regression analysis performed on the penetration experimental data under both 0.5 h and 24 h thermal storage conditions reveals that the developed models are highly significant (*p* < 0.05). This statistical verification confirms that the established response surface models can accurately capture and delineate the underlying relationships between the independent design variables and the penetration response.

The ANOVA results for the penetration response are tabulated in Table 9. The statistical outcomes indicate that the constructed models exhibit a high goodness-of-fit and minimal residual errors, rendering them highly reliable for predicting and analyzing the evolution laws of penetration. For the 0.5 h thermal storage condition, the significant variables of the model are identified as *A*, *B*, *C*, and $B^{2}$. This implies that within the linear (main) effects, the dosages of CR and aromatic oil all exert significant impacts on the penetration of the bitumen, with the aromatic oil dosage exhibiting an extremely significant dominance (*p* < 0.0001). Conversely, among the quadratic effects, only the CR dosage plays a significant role.

Upon extending the thermal storage period to 24 h, the regression model was re-established based on the corresponding experimental data. Initially, a full quadratic model including all linear, interaction, and quadratic terms was fitted. According to the ANOVA results (Table 9), the statistically insignificant terms (*p* > 0.05), including the interactions between SBS and CR dosages (*AB*) and between SBS and aromatic oil dosages (*AC*), were sequentially eliminated while preserving the model hierarchy. Consequently, the final reduced quadratic model retained the significant terms *B*, *C*, *BC*, $A^{2}$, $B^{2}$ and $C^{2}$.

Among the retained variables, the aromatic oil dosage (*C*) remained the most influential linear factor affecting penetration, indicating that the incorporation of aromatic oil effectively adjusted the consistency and rheological characteristics of the composite bitumen during prolonged thermal storage. Furthermore, the significant *BC* interaction demonstrates that the combined effect of crumb rubber and aromatic oil plays an important role in regulating the penetration response after thermal storage, while the significant quadratic terms ($A^{2}$, $B^{2}$ and $C^{2}$) reveal the existence of pronounced non-linear effects among the three modifiers. Based on these significant variables, the optimized reduced regression model was established as follows:

(9) $$Y_{1}\;=\;52.56-1.92A\;+\;2.36B\;+\;6.14C\;+\;6.96B^{2}$$

(10) $$Z_{1}=60.42+5.89B\;+10.24C\;+6.4\mathrm{BC}-4.11A^{2}+4.42B^{2}+4.27C^{2}$$

where $Y_{1}$ and $Z_{1}$ denote the predicted penetration values (0.1 mm) of the composite-modified bitumen after 0.5 h and 24 h of thermal storage, respectively.

**Figure 6:**
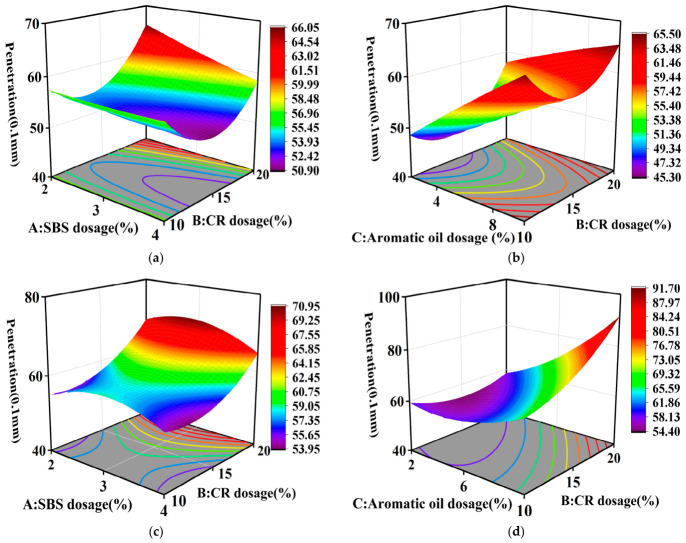
Effect of SBS, CR, and aromatic oil dosages on penetration under different storage conditions. (**a**) Interaction effect of SBS and CR dosages on penetration after 0.5 h of storage. (**b**) Interaction effect of aromatic oil and CR dosages on penetration after 0.5 h of storage. (**c**) Interaction effect of SBS and CR dosages on penetration after 24 h of storage. (**d**) Interaction effect of aromatic oil and CR dosages on penetration after 24 h of storage.

Table 9 shows that in the ANOVA for 0.5 h of thermal storage, the large difference between Adjusted R^2^ and Predicted R^2^ indicated model inaccuracy. However, after removing insignificant factors, the Adjusted R^2^ was 0.8111 and the Predicted R^2^ was 0.6758, indicating a good model fit. The response surface models for penetration constructed via Equations (9) and (10) are graphically illustrated in Figure 6. The response surface analysis indicates that the dosages of SBS, CR, and aromatic oil exert significant influences on the fluidity of the composite-modified bitumen, exhibiting pronounced interaction effects and non-linear response characteristics. Specifically, an increase in aromatic oil dosage leads to higher penetration, which is hypothesized to be due to the replenishment of light fractions that enhance system fluidity and reduce bulk viscosity. Conversely, SBS dosage tends to decrease penetration; this may be attributed to the formation of a continuous elastic network architecture that restricts the mobility of bitumen molecular chains [46]. However, this effect was not statistically significant in the current model. We incorporated studies by Airey et al. that CR dosage displays a non-linear pattern: while an optimal amount facilitates system stability, excessive loading is thought to induce particle agglomeration, which compromises matrix homogeneity and increases penetration [47,48].

A further comparative analysis between the response patterns under 0.5 h and 24 h thermal storage conditions reveals that prolonged high-temperature storage promotes the migration of light components and softens the system [49,50], which progressively intensifies the plasticizing effect of the aromatic oil on penetration. Simultaneously, it weakens the confinement capability of the SBS network over the CR particles due to thermodynamic polymer relaxation and phase separation [51,52], thereby complicating the internal structural evolution of the system and rendering the interaction effects and quadratic behaviors more pronounced. On the whole, judiciously controlling the aromatic oil dosage while sustaining the stability of the SBS network structure constitutes the cornerstone for achieving a synergistic equilibrium between the fluidity and high-temperature stability of the composite-modified bitumen.

**Figure 7:**
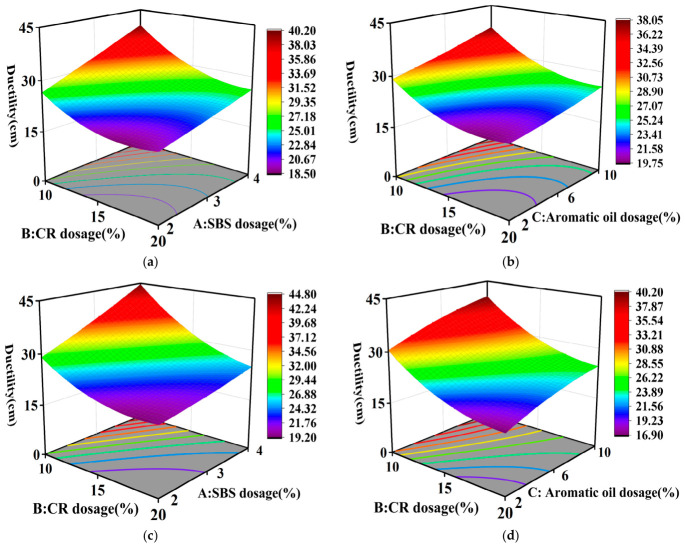
Effect of SBS, CR, and aromatic oil dosages on ductility under different storage conditions. (**a**) Interaction effect of SBS and CR dosages on ductility after 0.5 h of storage. (**b**) Interaction effect of aromatic oil and CR dosages on ductility after 0.5 h of storage. (**c**) Interaction effect of SBS and CR dosages on ductility after 24 h of storage. (**d**) Interaction effect of aromatic oil and CR dosages on ductility after 24 h of storage.

**Figure 8:**
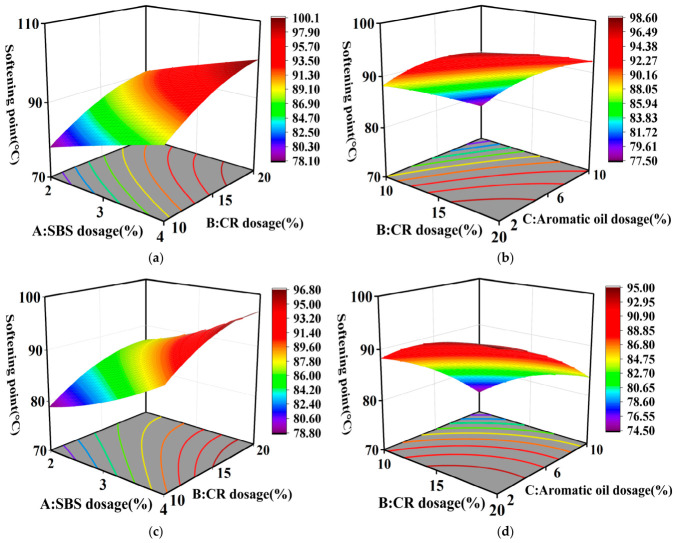
Effect of SBS, CR, and aromatic oil dosages on softening point under different storage conditions. (**a**) Interaction effect of SBS and CR dosages on softening point after 0.5 h of storage. (**b**) Interaction effect of aromatic oil and CR dosages on softening point after 0.5 h of storage. (**c**) Interaction effect of SBS and CR dosages on softening point after 24 h of storage. (**d**) Interaction effect of aromatic oil and CR dosages on softening point after 24 h of storage.

**Figure 9:**
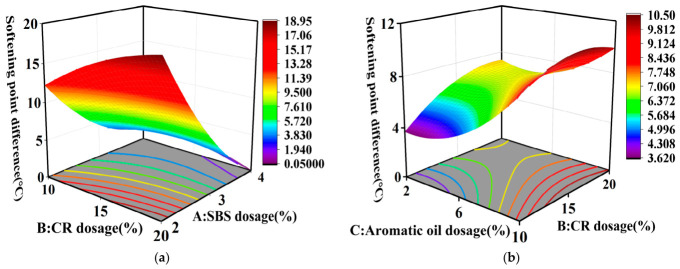
Response surface plots of the effects of SBS, CR, and aromatic oil dosages on softening point. (**a**) Interaction effect of SBS and CR dosages on softening point difference. (**b**) Interaction effect of aromatic oil and CR dosages on softening point difference.

In addition to penetration, ductility, softening point, and the softening point difference value were concurrently adopted as response metrics to comprehensively evaluate the performance profiles of the CR/SBS-composite-modified bitumen. The corresponding mathematical modeling, significance testing, and response surface analysis procedures follow an identical methodology to that established for the penetration model, and are thus omitted here for brevity. Table 10 lists the *p*-values, F-values, R^2^, Adjusted R^2^, and Predicted R^2^ of the ANOVA results for ductility, softening point, and softening point difference response. The final regression equations developed for these respective response targets are formulated in Equations (11)–(15), and their corresponding 3D response surface plots are presented in Figure 7, Figure 8 and Figure 9.

(11) $$Y_{2}=53.07+5.06A-5.49B+0.89C+0.15B^{2}$$

(12) $$Z_{2}=25.54+5.30A-7.28B+4.38C-2.52\mathrm{AB}+3.31B^{2}$$

(13) $$Y_{3}=45.16+4.76A\;+3.86B-1.07C-0.09B^{2}$$

(14) $$Z_{3}=70.21+5.06A\;+0.78B-1.57C$$

(15) $$Y_{4}=7.16-7.28A\;+1.93C\;+2.44A^{2}$$

where $Y_{2}$ and $Z_{2}$ denote the ductility values (cm) under the 0.5 h and 24 h thermal storage conditions, respectively; $Y_{3}$ and $Z_{3}$ represent the softening points (°C) after 0.5 h and 24 h thermal storage conditions, respectively; and $Y_{4}$ signifies the segregation indicator, expressed as the softening point difference (°C).

The response surface analysis outcomes demonstrate that SBS, CR, and aromatic oil dosages exert significant influences on the low-temperature ductility, high-temperature stability, and storage stability of the CR/SBS-composite-modified bitumen, exhibiting distinct non-linear interaction effects. Specifically, an increase in the SBS dosage significantly enhances the ductility and softening point while reducing the segregation softening point difference. Based on the research and experimental results of Laukkanen et al., it can be inferred that the continuous elastic network architecture formed by SBS not only bolsters the deformation resistance of the system and alleviates low-temperature stress concentrations, but also improves the compatibility and structural stability among the modifier components, establishing itself as a critical factor in uplifting the overall performance profiles of the composite system [53,54,55].

In contrast, Jeong et al.’s research showed that while an elevated CR dosage contributes to the formation of a spatial skeleton structure and enhances high-temperature stability, its absorption of light fractions escalates the matrix stiffness and restricts molecular chain mobility, thereby compromising the low-temperature ductility to some extent [56,57,58]. Furthermore, at higher CR loadings, its effects progressively manifest non-linear characteristics, as evidenced by a diminishing growth rate in high-temperature performance and a decline in matrix homogeneity. Aromatic oils primarily improve the system’s flexibility and workability by supplementing it with lighter components, and also have a certain plasticizing effect on ductility. However, excessive aromatic oil loading leads to the over-swelling and dilution of the polymer phase. Eltwati et al. believe that this dilution effect disrupts the continuity of the SBS elastic network and the CR skeleton, thereby reducing the macroscopic softening point and exacerbating thermodynamic instability and phase segregation [59,60].

A further comparative assessment of the response patterns under 0.5 h and 24 h thermal storage conditions reveals that prolonged hot-storage triggers the migration of light fractions and the relaxation of the polymer networks. This destabilizes the internal structure of the composite bitumen, thereby rendering the interactions among factors more pronounced and resulting in a certain degree of attenuation in both high-temperature performance and storage stability. On the whole, judiciously increasing the SBS dosage while strictly optimizing the CR and aromatic oil dosages constitutes the cornerstone for achieving a synergistic optimization of high- and low-temperature performance alongside the storage stability of the composite-modified bitumen.

#### 3.3.3. Prediction of Optimal Modifier Dosages

Based on the developed response surface models, the optimization objectives for the 0.5 h storage condition were defined as a penetration range of 50–70 (0.1 mm), maximum ductility and softening point, and minimum softening point difference. For the 24 h storage condition, the penetration range was adjusted to 60–70 (0.1 mm), while the remaining optimization objectives remained unchanged.

The optimization results indicated that the optimal formulation under the 0.5 h storage condition (Scheme I) consisted of 4 wt.% SBS, 10 wt.% CR, and 3.5 wt.% aromatic oil. Under the 24 h storage condition, the optimal formulation (Scheme II) consisted of 4 wt.% SBS, 13 wt.% CR, and 7.98 wt.% aromatic oil. The predicted response values corresponding to the optimal formulations obtained from the response surface optimization are summarized in Table 11. These two optimal validation formulations are located outside the original Box–Behnken experimental matrix, thus constituting an independent validation dataset.

### 3.4. Comprehensive Evaluation of Optimization Results Based on TOPSIS

**Figure 10:**
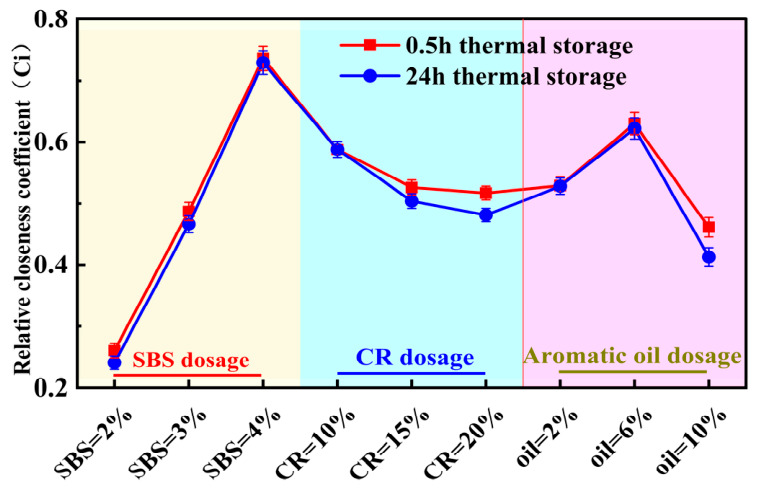
Effects of factor levels on the relative closeness coefficient.

Figure 10 presents the variation in the relative closeness coefficient ($C_{i}$) at different factor levels calculated using the entropy-weighted TOPSIS method. The effects of SBS dosage, CR dosage, and aromatic oil dosage on $C_{i}$ under the 0.5 h and 24 h storage conditions were comparatively evaluated, thereby elucidating the influence of each factor on the overall performance of the composite-modified bitumen.

As illustrated in Figure 10, similar variation patterns of the relative closeness coefficient ($C_{i}$) were observed under both the 0.5 h and 24 h storage conditions, indicating that the optimization results obtained from the response surface models were generally consistent. Nevertheless, a reduction in ($C_{i}$) was observed for all factors after extending the storage duration from 0.5 h to 24 h, suggesting that prolonged exposure to high temperatures adversely affected the overall performance of the composite-modified bitumen. The entropy-weighted TOPSIS results further revealed that a relatively high SBS dosage, a low CR dosage, and a low to moderate aromatic oil dosage were beneficial for improving the comprehensive performance of the bitumen. This combination contributed to a more favorable balance among high-temperature deformation resistance, low-temperature cracking resistance, and storage stability.

The calculated relative closeness coefficient ($C_{i}$) of Formulation I was 0.734, exceeding that of Formulation II (0.621), indicating that Formulation I exhibited superior comprehensive performance and a more favorable balance among the evaluated performance indicators.

Compared with subjective multi-criteria decision-making methods such as AHP, the entropy-weighted TOPSIS method determines indicator weights directly from the statistical characteristics of the experimental data, thereby minimizing subjective bias during the evaluation process. This makes it particularly suitable for simultaneously optimizing multiple performance indices of CR/SBS-composite-modified bitumen, including penetration, ductility, softening point, and softening point difference. Furthermore, although alternative weighting strategies were not investigated in the present study, Formulation I consistently outperformed Formulation II in both the comprehensive evaluation (Ci = 0.734 vs. 0.621) and the subsequent experimental validation. The difference between the two formulations reached approximately 18.2%, suggesting that moderate variations in indicator weights are unlikely to alter the final ranking. Nevertheless, a comprehensive sensitivity analysis considering equal-weight, AHP, or other weighting strategies will be conducted in future work to further evaluate the robustness of the proposed optimization framework.

### 3.5. Experimental Validation of Performance Metrics

To verify the reliability of the response surface optimization and the entropy-weighted TOPSIS evaluation results, composite-modified bitumen was prepared using the two optimized formulations, followed by performance validation tests. In addition to the response variables involved in the response surface models, including penetration, ductility, softening point, and softening point difference, elastic recovery and rotational viscosity at 180 °C were further evaluated to comprehensively assess the high-temperature performance, low-temperature performance, storage stability, and workability of the composite-modified bitumen. The results of three parallel tests are summarized in Table 12.

As shown in Table 12, the measured values generally agreed well with the response surface predictions, confirming the satisfactory predictive capability of the developed models within the investigated experimental domain. For Formulation I, the predicted penetration was 50 (0.1 mm), while the average measured penetration values were 52.4 and 54.1 (0.1 mm) after 0.5 h and 24 h of thermal storage, respectively. The corresponding deviations remained relatively small, indicating that the developed model accurately captured the performance evolution of the composite bitumen. Likewise, the predicted softening point (91.7 °C) and the measured values (91.8 °C after 0.5 h and 89.0 °C after 24 h) exhibited good agreement, further demonstrating the robustness of the regression model.

For Formulation II, however, relatively larger deviations were observed after prolonged thermal storage, exposing a critical methodological limitation of the applied response surface optimization. Although the predicted penetration was 60 (0.1 mm), the measured average value increased to 65.4 (0.1 mm) after 24 h, corresponding to a deviation of approximately 9.0%. Similarly, the measured ductility (34.4 cm) was lower than the predicted value (39 cm). Rather than a simple experimental error, this discrepancy fundamentally stems from the inherent limitations of conventional quadratic response surface models when applied to highly dynamic polymer systems. Static RSM polynomials are built upon the assumption of chemical and structural equilibrium. However, the relatively high aromatic oil dosage (7.98%) in Formulation II acts as a kinetic catalyst during prolonged (24 h) thermal storage. It significantly accelerates the continuous migration of light fractions and triggers severe secondary swelling and network relaxation of the crumb rubber. Consequently, the internal microstructure of the composite-modified bitumen transitions from a static state into a time-dependent dynamic evolution.

This highlights a major limitation of the present study: conventional static RSM models are inadequate for precisely predicting the long-term, kinetic-driven structural evolution of composite bitumen characterized by high oil content. The model accurately captures the initial pseudo-equilibrium state (e.g., 0.5 h) but fails to extrapolate the complex non-linear thermodynamic degradation occurring over 24 h. Therefore, while RSM remains a robust tool for preliminary screening and short-term optimization, its long-term predictive boundaries must be strictly recognized. This limitation fundamentally justifies why Formulation I (which inherently possesses higher network stability and less kinetic volatility due to its lower aromatic oil content) consistently outperformed Formulation II in the entropy-weighted TOPSIS evaluation. Future research must integrate time-dependent kinetic variables or machine learning algorithms to overcome the static constraints of traditional RSM methodologies.

Regarding elastic recovery, both formulations exhibited satisfactory elastic recovery capacities, indicating that the incorporation of SBS and CR contributed to the formation of an effective elastic network structure and enhanced the resistance of the binder to permanent deformation. In terms of workability, both formulations satisfied construction requirements. However, Formulation I exhibited a relatively lower rotational viscosity, which was more favorable for mixing and paving operations. By comparison, the higher dosages of CR and aromatic oil in Formulation II resulted in a slight increase in viscosity and a corresponding reduction in workability.

Considering the response surface optimization results, the entropy-weighted TOPSIS evaluation, and the experimental validation results, Formulation I demonstrated a more balanced combination of high-temperature stability, low-temperature flexibility, storage stability, and workability. Moreover, the performance indices of Formulation I showed smaller fluctuations, indicating better overall stability of the binder system. Therefore, the formulation containing 4 wt.% SBS, 10 wt.% CR, and 3.5 wt.% aromatic oil was identified as the recommended optimum composition for the CR/SBS-composite-modified bitumen developed in this study.

Compared with previous CR/SBS composite bitumen, the optimum formulation (4 wt.% SBS, 10 wt.% CR and 3.5 wt.% aromatic oil) achieved the target performance with a relatively lower CR dosage, benefiting from the high stiffness of the 60^#^ base bitumen and the compatibility enhancement provided by aromatic oil. Experimental validation further confirmed the reliability of the optimized formulation, with a penetration of 52.4–54.1 (0.1 mm), ductility of 33.1–37.6 cm, softening point of 89.0–91.8 °C, softening point difference below 1.2 °C (average 0.9 °C), elastic recovery of 88–89%, and rotational viscosity of 1.46–2.41 Pa·s, indicating a favorable balance among rutting resistance, crack resistance, storage stability, and workability.

## 4. Conclusions

To improve the engineering applicability of CR/SBS-composite-modified bitumen under extreme climate and long-distance transportation conditions in Xinjiang, a systematic optimization strategy integrating orthogonal experimental design, Box–Behnken response surface methodology (RSM), entropy-weighted TOPSIS comprehensive evaluation, and experimental validation was established. The preparation process, modifier dosages, and thermal storage evolution characteristics of the composite binder were comprehensively investigated. The major conclusions are summarized as follows:

1. The addition sequence of modifiers significantly affected the structural development and overall performance of the CR/SBS-composite-modified bitumen. Introducing SBS prior to CR promoted the formation of a relatively continuous polymer network during high-temperature shearing, which facilitated the subsequent swelling and uniform dispersion of CR particles [61]. Although the simultaneous addition strategy exhibited a slightly lower softening point difference, the SBS-first process achieved a superior balance among high-temperature performance, low-temperature ductility, and storage stability. Therefore, the SBS-first addition sequence was selected as the optimum preparation route.
2. The orthogonal experimental results demonstrated that the effects of processing parameters were highly dependent on both the evaluated performance index and the thermal storage duration [62]. During the 0.5 h of thermal storage, shear temperature exhibited the greatest influence on penetration (R = 16.1), shear speed dominated the softening point (R = 21.1), while shear duration showed the largest effects on ductility (R = 5.8) and storage stability (R = 24.4). After 24 h of thermal storage, the dominant factors shifted owing to the continuous swelling and structural evolution of the composite binder. Based on the combined evaluation of the mean response analysis and range analysis, the optimum processing parameters were identified as a shearing duration of 60 min, a shearing temperature of 185 °C, and a shearing speed of 4000 rpm, providing a balanced optimization of conventional performance and storage stability.
3. The response surface methodology successfully established statistically significant predictive models describing the relationships between SBS dosage, CR dosage, aromatic oil dosage, and the performance indices of the composite-modified bitumen. Most of the developed models exhibited excellent goodness-of-fit (R^2^ > 0.95), indicating high predictive capability under initial or short-term thermal conditions. The optimum modifier dosages predicted by the RSM under the short-term thermal storage condition were 4 wt.% SBS, 10 wt.% CR, and 3.5 wt.% aromatic oil, satisfying the target requirements of penetration, ductility, softening point, and softening point difference. By comparing the performance with that of actual prepared samples, it was found that the model can effectively map the influence of various factors on the performance indices. However, a major methodological limitation of conventional quadratic RSM was identified: it is inadequate for precisely forecasting the long-term (e.g., 24 h) kinetic-driven structural evolution of highly dynamic composite systems, particularly those with elevated aromatic oil contents. Therefore, while these empirical frameworks enable accurate performance forecasting within static equilibrium boundaries, future optimizations for prolonged engineering thermal aging must integrate time-dependent kinetic variables to overcome this fundamental static constraint.
4. Comparison of the response surface results obtained after 0.5 h and 24 h of storage revealed pronounced performance evolution during high-temperature storage. With increasing storage duration, penetration generally increased while the softening point decreased, indicating relaxation of the polymer network structure and migration of light components within the binder system [63]. These findings suggest that the influence of storage-induced evolution should be considered during the optimization and design of CR/SBS-composite-modified bitumen. From an engineering perspective, the proposed optimization strategy provides a practical methodology for simultaneously optimizing the preparation process and modifier formulation of CR/SBS-composite-modified bitumen. The proposed methodology can also serve as a reference for the optimization of other multi-component polymer-modified bitumen systems.
5. The entropy-weighted TOPSIS method effectively integrated multiple performance indicators into a comprehensive evaluation framework and avoided the potential bias associated with single-index optimization. The relative closeness coefficient of Formulation I (Ci = 0.734) was approximately 18.2% higher than that of Formulation II (Ci = 0.621), indicating that Formulation I achieved a better balance among high-temperature stability, low-temperature performance, storage stability, and workability. This method can provide a rigorous and objective evaluation of the overall performance balance of the composite system under multi-attribute conditions. Subsequent experimental validation further confirmed the reliability of the optimization results, with the measured penetration (52.4–54.1 0.1 mm), softening point (88.6–92.2 °C), softening point difference (0.7–1.2 °C), elastic recovery (88–89%), and rotational viscosity (1.30–2.47 Pa·s) showing good agreement with the predicted values.

This study focused on the optimization of preparation parameters, synergistic regulation of modifier dosages, and performance evolution during high-temperature storage of CR/SBS-composite-modified bitumen. Through the combined application of orthogonal experimental design and response surface methodology, the comprehensive performance of the composite system was systematically evaluated. The mechanisms regarding polymer swelling, network evolution, and phase compatibility were inferred primarily from macroscopic performance together with previous literature rather than direct microstructural evidence. Future research will further investigate the rheological properties, microstructural interaction mechanisms, and mixture performance of CR/SBS-composite-modified bitumen, with particular emphasis on its long-term service behavior and durability under the complex climatic conditions of Xinjiang. The outcomes are expected to provide both theoretical guidance and technical support for the engineering application and wider implementation of this composite-modified bitumen technology.

## Figures and Tables

**Table 1:** Fundamental properties of the base bitumen.

Properties		Chunfeng Tahe 60^#^
Penetration (100 g, 5 s, 25 °C)/0.1 mm		69.4
Softening point/°C		53.9
Ductility (25 °C, 5 cm/min)/cm		80.9
Solubility/%		99.8
Flash point/°C		278
Wax content/%		2.3
Density (25 °C)/(g/${\mathrm{cm}}^{3}$)		1.021
Residue after TFOT	Mass change/%	0.26
	Retained penetration/%	61
	Ductility (25 °C)/cm	27

**Table 2:** Technical properties of the SBS modifier.

Properties	Specifications	Test Results
Volatile matter/%	≤1.0	0.22
Ash content/%	≤0.25	0.13
Total styrene content/%	28~32	29.7
Tensile stress at 300% elongation/MPa	≥1.8	3.1
Elongation at break/%	≥520	663
Hardness (Shore A)	≥68	75.4
Number-average molecular weight/(g/mol)	8–13 × 10^4^	10.5 × 10^4^
Weight-average molecular weight/(g/mol)	15–25 × 10^4^	19 × 10^4^
Polydispersity index	1.5–2.2	1.9

**Table 3:** Chemical specifications of the crumb rubber.

Properties	Specifications	Test Results
Ash content/%	≤8	7.6
Acetone extract/%	≤16	7
Rubber hydrocarbon content/%	≥48	57
Carbon black content/%	≥28	28.4

**Table 4:** Technical specifications of the cross-linking stabilizer (elemental sulfur).

Properties	Specifications	Test Results
Active ingredient content (%)	≥99.0	99.4
Apparent density/(g/${\mathrm{cm}}^{3}$)	0.9–1.1	0.98
Melting point/°C	≥120	124
Fineness (residue on an 80-mesh sieve)/%	≤2	0.8
Moisture/%	≤0.1	0.04
Added amount/%	1–2	1.5

**Table 5:** Orthogonal experiment factors and levels.

Factor	Shearing Duration (min)	Shearing Temperature (°C)	Shearing Speed (rpm)
Level 1	30	185	3000
Level 2	60	190	4000
Level 3	90	195	5000

**Table 6:** Orthogonal experimental design matrix.

No	Shearing Duration (min)	Shearing Temperature (°C)	Shearing Speed (rpm)
1	30	185	3000
2	60	190	4000
3	90	195	5000
4	30	190	5000
5	60	195	3000
6	90	185	4000
7	30	195	4000
8	60	185	5000
9	90	190	3000

**Table 7:** Experimental factors and levels for response surface methodology (RSM) design.

Coded Level	A: SBS Dosage/wt.%	B: CR Dosage/wt.%	C: Aromatic Oil Dosage/wt.%
−1	2	10	2
0	3	15	6
1	4	20	10

**Table 8:** Results of response surface methodology.

No	SBS (%)	CR (%)	Aromatic Oil (%)	0.5 h Thermal Storage				24 h Thermal Storage		
				Penetration (0.1 mm)	Ductility (cm)	Softening Point (°C)	Softening Point Difference (°C)	Penetration (0.1 mm)	Ductility (cm)	Softening Point (°C)
1	4	20	6	59.6	27.1	100.5	0.5	63.2	26.5	97.5
2	3	20	10	64.5	25.6	91.2	9.5	93.2	24.4	82.1
3	3	15	6	55	24.5	88.5	9	65.7	25.7	86.9
4	3	15	6	54.1	27.5	92.1	4.2	59.3	28.9	89.7
5	2	10	6	56.2	25.6	77.7	11.8	56.5	27.7	78.1
6	3	10	10	67	38	78	8.9	65.9	40.8	74.8
7	2	20	6	67.6	18.8	91.8	19.1	67.3	19.1	87.8
8	4	15	2	42.5	24.1	100.6	2.5	47.7	23.4	99.2
9	4	10	6	55.9	40.9	87	1.8	55.9	45.2	88.1
10	4	15	10	59	33.9	91.5	4.5	72.1	35.7	88.1
11	2	15	10	57.5	23	82.5	21.4	69.1	23.1	77.5
12	3	10	2	49.5	29.4	89.1	4.1	57.8	30.8	89.8
13	3	15	6	50.5	23.2	94	6.7	59.7	25.1	91.6
14	3	15	6	50.3	21.5	91.6	9.8	55.9	23.9	86.6
15	3	20	2	55.8	20.4	98.1	7.1	59.5	16.3	94.6
16	2	15	2	51.1	18.1	89.5	15.2	53.4	18.5	89
17	3	15	6	53	24.9	91.9	5.1	61.5	25.6	89.5

**Table 9:** Analysis of variance (ANOVA) for penetration.

Factor	0.5 h Thermal Storage			24 h Thermal Storage		
	Sum of Squares	*F*	*p*	Sum of Squares	*F*	*p*
Model	641.10	14.10	0.0011	1531.63	17.79	0.0005
A	29.64	5.87	0.0459	6.85	0.7156	0.4256
B	44.65	8.84	0.0207	277.30	28.99	0.0010
C	301.35	59.64	<0.0001	838.45	87.65	<0.0001
AB	14.82	2.93	0.1305	3.06	0.3202	0.5892
AC	25.50	5.05	0.0595	18.92	1.98	0.2024
BC	19.36	3.83	0.0912	163.84	17.13	0.0044
$A^{2}$	0.3420	0.0677	0.8022	71.12	7.44	0.0295
$B^{2}$	203.96	40.37	0.0004	82.07	8.58	0.0221
$C^{2}$	0.4867	0.0963	0.7653	76.59	8.01	0.0254
$R^{2}$	0.9477			0.9581		
Adjusted $R^{2}$	0.8805			0.9043		
Predicted $R^{2}$	0.5448			0.7926		

**Table 10:** ANOVA test results for quadratic response surface models.

Response Value	F	*p*	$R^{2}$	Adjusted $R^{2}$	Predicted $R^{2}$
$Y_{2}$	18.52	0.0004	0.9597	0.9079	0.8048
$Z_{2}$	31.06	<0.0001	0.9756	0.9442	0.8257
$Y_{3}$	25.35	0.0002	0.9702	0.9320	0.8553
$Z_{3}$	19.52	0.0004	0.9617	0.9124	0.7489
$Y_{4}$	16.88	0.0006	0.9559	0.8993	0.9058

**Table 11:** Predicted response values of the optimal formulations.

Scheme	SBS (wt.%)	CR (wt.%)	Aromatic Oil (wt.%)	Penetration	Ductility	Softening Point	Softening Point Difference
I	4	10	3.5	50	36.5	91.7	1.7
II	4	13	7.98	60	39	89.5	3.2

**Table 12:** Experimental validation results of the optimized formulations.

Formulation	Storage Time (h)	Penetration (0.1 mm)	Ductility (cm)	Softening Point (°C)	Softening Point Difference (°C)	Elastic Recovery (%)	Rotational Viscosity (Pa·s)
I	0.5	51.8	32.5	92.2	0.7	89	2.474
		53.1	33.6	91.3	1.2	89	2.335
		52.4	33.1	91.8	0.9	89	2.413
	24	53.8	36.5	89.3	0.7	88	1.612
		54.3	38.5	88.6	1.2	88	1.299
		54.1	37.8	89	0.9	88	1.465
II	0.5	54.3	32.5	90.4	2.2	90	2.562
		55.8	33.3	90.8	2.8	90	2.537
		55.3	33	90.1	2.4	90	2.514
	24	64.9	33.3	90.3	2.2	89	1.713
		65.9	35.5	89.3	2.8	88	1.568
		65.3	34.5	89.6	2.4	89	1.682

## Data Availability

The original contributions presented in this study are included in the article. Further inquiries can be directed to the corresponding author.
